# Memophenol^TM^ Prevents Amyloid-β Deposition and Attenuates Inflammation and Oxidative Stress in the Brain of an Alzheimer’s Disease Rat

**DOI:** 10.3390/ijms24086938

**Published:** 2023-04-08

**Authors:** Daniela Impellizzeri, Mario Tomasello, Marika Cordaro, Ramona D’Amico, Roberta Fusco, Ali S. Abdelhameed, Uwe Wenzel, Rosalba Siracusa, Vittorio Calabrese, Salvatore Cuzzocrea, Rosanna Di Paola

**Affiliations:** 1 Department of Chemical, Biological, Pharmaceutical and Environmental Sciences, University of Messina, Viale F. Stagno D’Alcontres 31, 98166 Messina, Italy; dimpellizzeri@unime.it (D.I.); rdamico@unime.it (R.D.); rfusco@unime.it (R.F.); salvator@unime.it (S.C.); 2 Department of Biomedical and Biotechnological Sciences, University of Catania, Via Santa Sofia 97, 95123 Catania, Italy; mario.tomasello@studium.unict.it (M.T.); calabres@unict.it (V.C.); 3 Department of Biomedical, Dental and Morphological and Functional Imaging, University of Messina, Via Consolare Valeria, 98125 Messina, Italy; marika.cordaro@unime.it; 4 Department of Pharmaceutical Chemistry, College of Pharmacy, King Saud University, Riyadh 11451, Saudi Arabia; asaber@ksu.edu.sa; 5 Institut für Ernährungswissenschaft, Justus Liebig Universitat Giessen, 35390 Giessen, Germany; uwe.wenzel@ernaehrung.uni-giessen.de; 6 Department of Veterinary Sciences, University of Messina, Viale SS Annunziata, 98168 Messina, Italy; dipaolar@unime.it

**Keywords:** Nrf2, oxidative stress, neuroinflammation, brain damages, Memophenol^TM^

## Abstract

Alzheimer’s disease (AD) is the most common cause of dementia, and its prevalence rises with age. Inflammation and altered antioxidant systems play essential roles in the genesis of neurodegenerative diseases. In this work, we looked at the effects of Memophenol^TM^, a compound rich in polyphenols derived from French grape (*Vitis vinifera* L.) and wild North American blueberry (*Vaccinium angustifolium* A.) extracts, in a rat model of AD. Methods: For 60 days, the animals were administered with AlCl_3_ (100 mg/kg, orally) and D-galactose (60 mg/kg, intraperitoneally), while from day 30, Memophenol^TM^ (15 mg/kg) was supplied orally for 30 consecutive days. AlCl_3_ accumulates mainly in the hippocampus, the main part of the brain involved in memory and learning. Behavioral tests were performed the day before the sacrifice when brains were collected for analysis. Results: Memophenol^TM^ decreased behavioral alterations and hippocampus neuronal degeneration. It also lowered phosphorylated Tau (p-Tau) levels, amyloid precursor protein (APP) overexpression, and β-amyloid (Aβ) buildup. Furthermore, Memophenol^TM^ reduced the pro-oxidative and pro-inflammatory hippocampus changes caused by AD. Our finding, relevant to AD pathogenesis and therapeutics, suggests that Memophenol^TM^, by modulating oxidative and inflammatory pathways and by regulating cellular brain stress response mechanisms, protects against the behavioral and histopathological changes associated with AD.

## 1. Introduction

Alzheimer’s disease (AD) is among the most common senile dementias that occur in later life, representing a leading cause of disability and death in the elderly. The lengthening of life has led to the aging of the world population; therefore, it is estimated that every 20 years, the number of people affected by AD will double from the current number of 26.6 million to 106.8 million by 2050 [1]. From a neuropathological point of view, AD is characterized by the deposition of β-amyloid (Aβ) peptides, the formation of neurofibrillary tangles, cerebral angiopathy, astrocyte and microglia activation, and neuronal loss, leading to progressive cognitive and memory impairment [2]. According to the most accredited hypothesis, the key pathogenetic event responsible for the degeneration of neurons and for morphological, functional, and cognitive modifications is an excessive formation or accumulation of amyloid-genetic peptides [3].

Furthermore, the Aβ peptide would seem to have a crucial role in biological fluids and in the cells they contain [4]. Human erythrocytes, for example, show a loss of oxygen-dependent metabolic modulation following exposure to the Aβ peptide [5,6].

Neuroinflammation and oxidative stress have been shown to be significant contributors to AD disease progression and chronicity [7,8,9]. Therefore, the main strategies that should be used to prevent or treat the disease should be aimed at reducing inflammation, oxidative imbalance, and the accumulation of Aβ in the brain. AD still remains an incurable disease. In fact, despite the considerable progress made in recent years by biomedical research, there is still no therapeutic intervention that has been shown to be capable of reversing or stopping the underlying pathological process of this disease, but rather only of acting on the symptoms [10,11]. Furthermore, considering that many treatments currently used cause significant side effects, it is not surprising that today much attention is paid to the positive aspects that foods have, among other things, also on increasingly widespread neurological diseases in the modern world. Precisely because neurodegenerative diseases are characterized by a long preclinical phase, it is possible to go and act with nutrition to prevent the onset or slow down the progression of the disease. In fact, nutrients act as “nutraceuticals”, that is, as food principles that have beneficial effects on health [12,13,14,15,16].

Epidemiological studies have shown that consuming diets rich in anti-inflammatory and antioxidant agents, such as those found in fruits and vegetables, can reduce the risk of developing age-related neurodegenerative diseases [17,18,19,20]. Previous studies have revealed that daily consumption of grape and blueberry juice for a total of 12 weeks improved memory in elderly volunteers [21,22]. In another study, it was shown that the combined consumption of a grape and blueberry extract for 8 weeks was able to prevent memory decline in old mice [23]. The neuroprotective effects of these compounds seem to be linked to the high content of polyphenols, in particular of monomers and proanthocyanidins of flavanols, which have also been shown to have a protective effect in a study on cognitive impairment and cerebral aging induced by D-galactose. Based on the notions learned from the literature, in our study we evaluated the effect of the integration of a compound consisting of extracts of French grape and wild North American blueberry known as Memophenol^TM^ on the molecular and cognitive alterations of AD in the aluminum-induced rat model.

## 2. Results

### 2.1. Effects of Memophenol^TM^ on Behavioral and Histological Alterations

AD is a disease characterized by cognitive alterations which are in turn due to changes that occur in neurons, especially in the hippocampus. For this reason, we investigated the effect of Memophenol^TM^ both on the behavior and on the tissue alteration of the CA1 region of the hippocampus in AlCl_3_-treated rats.

**Figure 1:**
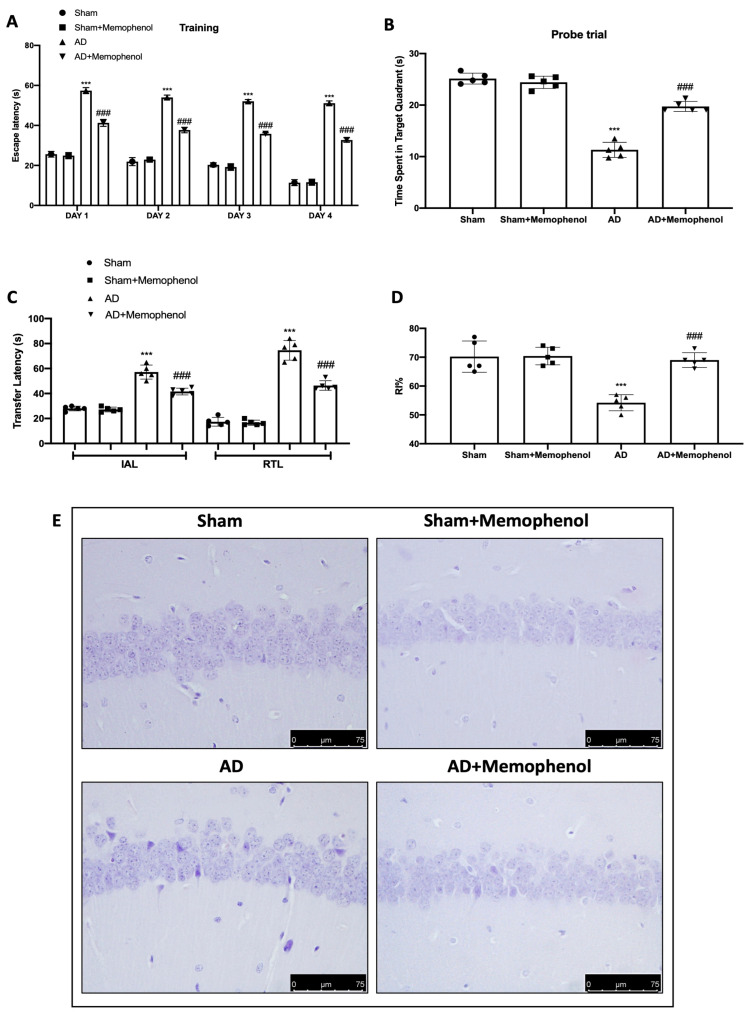

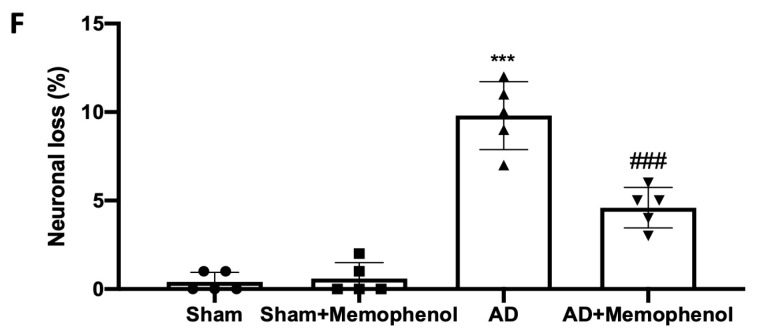
The administration of Memophenol^TM^ reduced behavioral and hippocampal alterations. MWM test: training (**A**); probe trial (**B**); EPM test (**C**); NOR test (**D**); histological analysis: Sham, Sham + Memophenol, AD, AD + Memophenol (**E**); quantification of necrotic neurons (**F**). Scale bar 75 μm. For the behavioral and histological investigations, n = 5 rats were used from each group and for each analysis. A *p*-value less than 0.05 was regarded as significant. *** *p* < 0.001 versus Sham, ### *p* < 0.001 versus AD.

On day four of the Morris Water Maze (MWM) test’s training period, when compared to day one, the animals in all groups demonstrated a diminishing trend in escape latency time (Figure 1A). Memophenol^TM^ increased animal persistence in the target quadrant in the searching experiment, indicating an increase in memory consolidation as compared to the AD group (Figure 1B). The Memophenol^TM^-treated rats showed a decrease in the time of transfer latency in initial acquisition latency (IAL) and retention transfer latency (RTL) in the Elevated Plus Maze (EPM) test, indicating an improvement in memory retention when compared to the AD group (Figure 1C). The Memophenol^TM^ treatment significantly enhanced the recognition index % (RI) in the novel object recognition (NOR) test, indicating an improvement in cognitive function as compared to the AD group (Figure 1D). The control group’s brain samples revealed normal tissue organization in the CA1 hippocampus region (Figure 1E,F). Tissues from the AD group, on the other hand, exhibited substantially more severe neuronal degeneration, with fewer dark basophilic neurons in the CA1 hippocampal pyramidal and polymorphic layers (Figure 1E,F). Memophenol^TM^ treatment greatly decreased AlCl_3_-induced CA1 neuronal degeneration (Figure 1E,F). Furthermore, both behavioral (Figure 1A–D) and histological analysis (Figure 1E,F) revealed no difference between the Sham and Sham + Memophenol^TM^ groups; hence, a molecular study on the control animals administered with Memophenol^TM^ was omitted.

### 2.2. Effects of Memophenol^TM^ Treatment on Aβ Deposition and APP and p-Tau Over-Expression

**Figure 2:**
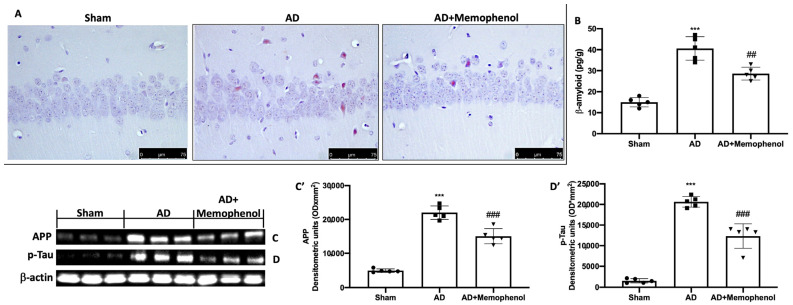
Memophenol^TM^ reduced Aβ deposition in AD rats. Congo red staining (labeled amyloidosis) in the hippocampus (**A**); ELISA analysis for β-amyloid levels (**B**); Western blot analyses for APP (**C**) and p-Tau (**D**) with related densitometric analysis (**C’**,**D’**); scale bar 75 μm. For the Western blot and ELISA investigations, n = 5 rats from each group and for each analysis were employed. A *p*-value of less than 0.05 was considered significant. *** *p* < 0.001 versus Sham, ## *p* < 0.01 versus AD, ### *p* < 0.001 versus AD.

To demonstrate that Memophenol^TM^ had action on amyloidosis typical of AD, we performed Congo red staining. The staining results showed that more Aβ deposits were present in the hippocampus of the AD group animals, while these deposits were significantly reduced after Memophenol^TM^ treatment (Figure 2A). The result was further confirmed by an analysis of Aβ levels with an ELISA kit as presented in Figure 2B. Furthermore, we wanted to evaluate whether Memophenol^TM^ was also able to act on two other specific markers of AD disease such as APP and p-Tau. APP (Figure 2C,C’) and p-Tau (Figure 2D,D’) expression levels were higher in the hippocampi from the AD group compared to the Sham group. Administration of Memophenol^TM^ considerably lowered both levels (Figure 2C,C’,D,D’).

### 2.3. Memophenol^TM^ Treatment Effects on Oxidative Hippocampal Modifications

**Figure 3:**
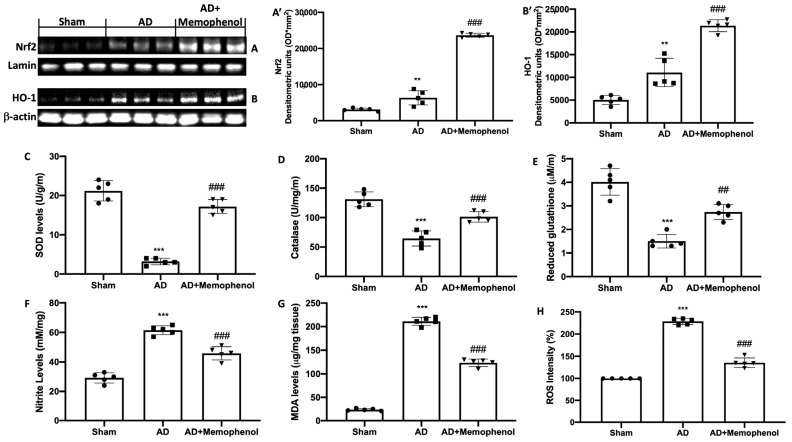
Memophenol^TM^ administration decreased pro-oxidative modifications in the hippocampus. Western blot analysis of Nrf2 expression (**A**) and HO-1 (**B**) with related densitometric analysis (**A’**,**B’**), and biochemical analysis of SOD levels (**C**), CAT activity (**D**), GSH levels (**E**), nitrite levels (**F**), MDA levels (**G**), and ROS levels (**H**). For both analyses, n = 5 rats from each group and for each analysis were employed. A *p*-value of less than 0.05 was considered significant. ** *p* < 0.01 versus Sham, *** *p* < 0.001 versus Sham, ## *p* < 0.01 versus AD, ### *p* < 0.001 versus AD.

It is known that oxidative stress is an important risk factor for this pathology. In this regard, we wanted to evaluate the antioxidant activity of Memophenol^TM^ by Western blot analysis and biochemical tests on the hippocampus. Increased nuclear factor erythroid 2–related factor 2 (Nrf2) expression was found in the hippocampi of Memophenol^TM^-treated rats as compared to the AD and Sham groups (Figure 3A,A’). The same trend was also observed for heme oxygenase-1 (HO-1) (Figure 3B,B’). Furthermore, biochemical analysis revealed that Memophenol^TM^-treated rats had improved antioxidant defenses. Superoxide dismutase (SOD) levels (Figure 3C), catalase (CAT) activity (Figure 3D), and glutathione (GSH) levels (Figure 3E) were all significantly higher in the Memophenol^TM^ group than in the AD group. In contrast, nitrite (Figure 3F), lipid peroxidation (MDA) (Figure 3G), and reactive oxygen species (ROS) (Figure 3H) levels in the AD group were considerably higher than in the Sham group. Treatment with Memophenol^TM^ significantly reduced nitrite levels, lipid peroxidation, and ROS levels in the hippocampus.

### 2.4. Memophenol^TM^ Treatment Effects on Pro-Inflammatory Markers

**Figure 4:**
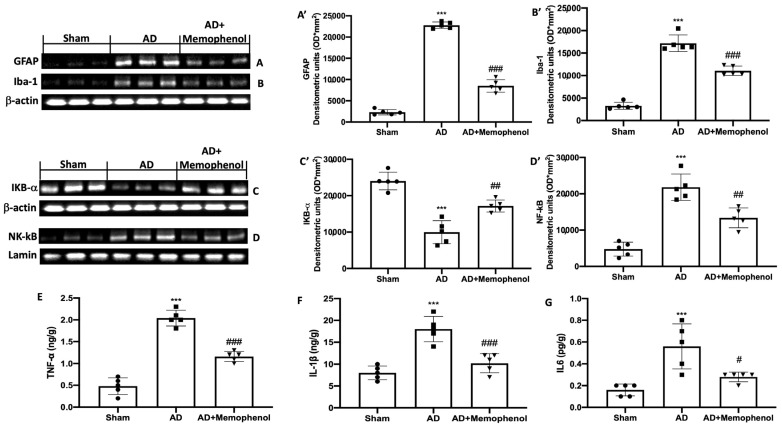
The effects of Memophenol^TM^ on the activation of astrocytes and microglia and on the NF-κB pathway following AlCl_3_ injection. Western blot analyses of GFAP (**A**), Iba-1 (**B**), IkB-α (**C**), and NF-κB (**D**) expression with related densitometric analysis (**A**’–**D**’); ELISA kit of TNF-α (**E**), IL-1β (**F**), and IL-6 (**G**) levels. For the Western blot and ELISA analyses, n = 5 rats from each group and for each analysis were employed. A *p*-value of less than 0.05 was considered significant. *** *p* < 0.001 versus Sham, # *p* < 0.05 versus AD, ## *p* < 0.01 versus AD, ### *p* < 0.001 versus AD.

Another fact implicated in the progression of AD is neuroinflammation. For this reason, in addition to the antioxidant action of Memophenol^TM^, we also wanted to investigate its anti-inflammatory properties. Western blot investigations on hippocampus tissue for glial fibrillary acid protein (GFAP) and ionized calcium-binding adaptor molecule 1 (Iba-1) expression were used to examine astrocyte and microglial cell activity in connection to AD. The GFAP and Iba-1 expressions were low in the Sham group but significantly higher in the AD-treated rats. Memophenol^TM^ treatment reduced the elevated expression of GFAP and Iba-1 under these conditions (Figure 4A,A’ for GFAP and Figure 4B,B’ for Iba-1). In addition, the Western blot analysis revealed a significant downregulation of the NF-κB pathway, which was activated by AlCl_3_ injection. The AD rat samples c α (IkB-α) expression in the cytoplasm (Figure 4C,C’), and enhanced NF-κB nuclear localization (Figure 4D,D’). Treatment with Memophenol^TM^ boosted IkB-α expression while restoring NF-κB expression to baseline levels. Memophenol^TM^ administration also lowered tumor necrosis factor-α (TNF-α) (Figure 4E), interleukin-1β (IL-1β) (Figure 4F), and interleukin-6 (IL-6) (Figure 4G) levels, which were elevated in the AD group due to the activity of the NF-κB pathway.

## 3. Discussion

Many chronic diseases, including Alzheimer’s, are determined by both heredity and environment. The genetic abnormalities of the APP and presenilin genes account for just 5% of the overall number of AD patients (familial instances), but the majority of AD patients are most likely due to environmental and other genetic variables affecting Aβ clearance [3]. Major environmental influences are likely to include an excess or deficit of dietary ingredients with bioactivity in key pathways that are taken on a regular basis. Our understanding of how food and drink might potentially impact the development of AD will aid in the development and implementation of medicines to battle this deadly illness. Memophenol^TM^ is derived from French grape and wild blueberry extracts and has a specific mix of essential polyphenols that has been clinically demonstrated to increase learning and memory functions. These polyphenols appear to have a threefold impact for a synergistic protective effect on the brain, acting in two ways: by enhancing both neurogenesis and sympathetic plasticity. In this regard, a clinical study was conducted on 215 elderly people which demonstrated that chronic integration of Memophenol^TM^ improves both short- and long-term memory [24]. In addition, a preclinical study showed that supplementation with a diet rich in polyphenols derived from grape and blueberry extracts prevented spatial locomotor impairments in middle-aged mice [23]. Another preclinical study was also conducted which highlighted the ability of polyphenol-rich grape and blueberry extracts to attenuate cognitive decline and improve neuronal function in aged mice [25]. In this study, an increase in the neurogenerative process was observed in elderly mice that assumed a diet rich in polyphenols extracted from grapes and blueberries compared to mice of the same age that were not supplemented with the same diet. Furthermore, some of the polyphenols included in the extract have been found in the brain in their native forms or as metabolites. This indicates that polyphenols may act directly centrally, while they may impact mouse survival through a potential systemic effect [25]. Given the observed effects on memory, it was decided to test this compound in an animal model of AD. In particular, in this study, we looked at the impact of Memophenol^TM^ supplementation on reactive oxygen species and the inflammatory processes that define AD. Several studies have demonstrated the role of oxidative stress in the growth and development of disease. According to recent research, AD has a latent phase before symptoms appear and a diagnosis is obtained. When compared to healthy individuals, the development of AD is preceded by a moderate cognitive impairment phase with a minor increase in Aβ deposition but with a considerable oxidative imbalance [26,27]. A significant amount of research has revealed that excessive ROS generation causes neuronal death and other pathological alterations in AD [28,29]. Oxidative damage is associated with the abnormal accumulation of Aβ and the overexpression of APP and p-Tau [8,30]. Elevated APP levels are linked to decreased hippocampus neurogenesis and, as a result, poorer cognitive function [31,32]. In fact, some evidence suggests that hippocampal plasticity is connected to memory consolidation, learning, and cognitive function [33,34]. Animal studies have conclusively shown that AlCl_3_ neurotoxicity is involved in the development of neurodegenerative illnesses such as Alzheimer’s. By aggregating Tau proteins, it enhances the development of Aβ protein plaques in the brain. AlCl_3_ has also been connected to the neurodegeneration and modifications associated with aging. AlCl_3_ toxicity, according to [35], is produced by increased ROS release, which causes oxidative damage in the hippocampus. Although aluminum is not a transition metal and cannot catalyze redox reactions, AlCl_3_ can induce neurotoxicity by generating free radicals [36,37]. Aluminum ions have a strong affinity for bio-membranes and can exacerbate the cellular oxidative environment by strengthening transition metal pro-oxidant properties [38]. It has also been associated with mitochondrial function impairment in vitro and in vivo, as well as impairment of the antioxidant defense system, which may lead to the development of oxidative stress [39,40,41]. AlCl_3_ treatment mostly accumulates in the hippocampus, which is known to be particularly vulnerable to AD and to play an important role in learning and memory processes [42]. For these reasons, hippocampus tissue was subjected to histological, biochemical, and molecular studies. Memophenol^TM^ inhibited the course of AD by lowering Tau hyperphosphorylation, APP levels, and Aβ buildup. From a behavioral standpoint, it significantly decreased cognitive deficits. Histologically, it decreased the chronic hippocampus neuron loss and degeneration features of AD. These behavioral and histological effects might be attributed to the molecular characteristics of Memophenol^TM^. It boosted cellular defenses against ROS by boosting the Nrf2/HO-1 pathway. Nrf2 oversees genes that code for endogenous antioxidant enzymes, redox balance factors, and stress response proteins [28,43,44]. It specifically stimulated phase II detoxification enzymes such as CAT, SOD, and GSH. Furthermore, Memophenol^TM^ lowered nitrite levels, lipid peroxidation, and ROS levels that were elevated by AD [45]. These anti-oxidative stress effects resulted in a decrease in the pro-inflammatory macroenvironment. We found that reducing the activity of the NF-κB pathway has significant anti-inflammatory properties. NF-κB is a key transcription factor in pro-inflammatory signaling [46,47]. In healthy settings, NF-κB is bound to its inhibitor IkB-α and is sequestered within the cytoplasm [48]. The inhibitor is destroyed during inflammation, and NF-kB translocates into the nucleus to encode pro-inflammatory proteins [49]. Our findings demonstrated restored cytoplasmic levels of IkB-α and decreased NF-κB nuclear expression of associated target pro-inflammatory mediators such as TNF-α, IL-1β, and IL-6. The aggregation of Aβ plaques can also lead to the activation of astrocytes and microglia, which are not only secondary players in pathological processes, but seem to contribute to synaptic and neuronal loss and to the accumulation of pathogenic proteins even in the early stages of disease [50,51,52]. Our treatment with Memophenol^TM^ also demonstrated the ability to reduce AlCl_3_-induced astrogliosis and microgliosis by reducing hippocampal GFAP and Iba-1 expression.

## 4. Materials and Methods

### 4.1. Tested Product: Memophenol^TM^

Memophenol^TM^ provided by the company Activ’Inside is a standardized polyphenol-rich extract from grapes and blueberries; it is abundant in bioavailable flavonoids and can operate locally on cognitive functioning by bridging the blood–brain barrier. Total flavonoids (flavan-3-ols, flavonols, and anthocyanins): >43%, flavan-3-ols monomers; ≥20%, oligomers (DP ≤ 4); ≥22%, flavonols (quercetin and glycosylated derivatives); ≥0.15%, anthocyanins: ≥0.10%).

### 4.2. Animals

Male Wistar rats (Envigo, Milan, Italy) were used (age: six to eight weeks, weight: 250–280 g). The animals were kept in a confined space and fed standard rodent chow (Envigo, Teklad Rodent Diet T.2018.12) and water. The study was approved by the University of Messina’s Review Board for Animal Care (OPBA). All animal experiments were conducted in accordance with new Italian legislation (D.Lgs 2014/26), EU legislation (EU Directive 2010/63), and the ARRIVE guidelines.

### 4.3. Experimental Protocol

Aluminum (AlCl_3_) is a popular AD model [53]. For 60 days the rats were treated with AlCl_3_ (100 mg/kg, orally) and D-galactose (60 mg/kg, intraperitoneally) [35,54,55].

#### Experimental Groups

The rats were randomly divided into the following groups (n = 20 for each group):

- Sham group: saline was administered to the rats;
- Sham + Memophenol^TM^ group: saline was administered to the rats, and Memophenol^TM^ (15 mg/kg) was administered orally for 30 consecutive days;
- AD group: as previously mentioned, the rats were treated with AlCl_3_ (100 mg/kg, orally) and D-galactose (60 mg/kg, intraperitoneally) for 60 days;
- AD + Memophenol^TM^ group: as previously documented, the rats were treated with AlCl_3_ (100 mg/kg, orally) and D-galactose (60 mg/kg, intraperitoneally) for 60 days, and Memophenol^TM^ (15 mg/kg) was supplied orally by gavage for 30 consecutive days.

The dose of Memophenol^TM^ was based on previous studies performed in the laboratory in which the compound was administered in increasing doses for 3 months. Behavioral test training was completed prior to the experiment’s conclusion date. The animals were sacrificed at the end of the trial after behavioral changes were examined. Brain tissues were collected for further analysis.

### 4.4. Behavioral Assessment

#### 4.4.1. MWM

To assess spatial learning and memory consolidation, the MWM test was used [18,56]. The percentage of distance walked and the amount of time spent in the target quadrant were both recorded.

#### 4.4.2. EPM

The EPM exam was used to assess memory-related activities. The behavioral test was carried out as previously reported [57,58].

#### 4.4.3. NOR

The NOR test was used to measure cognitive function abnormalities induced by Alzheimer’s disease. The behavioral test was carried out as previously reported [59]. The RI was used to record the time spent studying the unfamiliar object. It was calculated by dividing the amount of time spent examining a novel object (TN) by the amount of time spent exploring a familiar object (TF), [RI = TN/(TN + TF)]. An RI % larger than 50% implies more time spent finding the TN, whereas an RI % less than 50% indicates more time spent investigating the TF [60].

### 4.5. Histological Analysis and Congo Red Staining

Brain samples were taken and processed, and slices (7 μm) were cut into longitudinal sections and stained with hematoxylin and eosin (H&E) [61,62]. The necrosis percentages of necrotic neurons out of total neurons were manually counted along the ipsilateral hippocampus CA1 region [63]. Furthermore, some sections, after being deparaffinized and dehydrated by alcohol gradients, were stained with Highman Congo red staining solution for 5–10 min as described by Xia Zhao et al. [61]. An experienced histopathologist examined the sections under a Leica DM6 microscope (Leica Microsystems SpA, Milan, Italy) with a motorized stage and Leica LAS X Navigator 7.31 software (Leica Microsystems SpA, Milan, Italy).

### 4.6. Western Blot Analysis

Western blots on the hippocampi were performed as previously described [46,64]. Specific primary antibodies were used, such as anti-IkB-α (Santa Cruz Biotechnology, sc-1643), anti-NF-κB p65 (Santa Cruz Biotechnology, sc-8008), anti-Nrf2 (Santa Cruz Biotechnology, sc-36594), anti-HO-1 (Santa Cruz Biotechnology, sc-136960), anti-p-Tau (Santa Cruz Biotechnology, sc-32275), anti-APP (Santa Cruz Biotechnology, sc-32277), anti-GFAP (Cell Signaling Technology, Danvers, MA, USA), and anti-Iba1 (Santa Cruz Biotechnology, sc-32725), and they were mixed in a 5% w/v non-fat dried milk solution and were incubated at 4 °C overnight. The blots were then incubated for 1 h at room temperature with a peroxidase-conjugated bovine anti-mouse IgG secondary antibody or a peroxidase-conjugated goat anti-rabbit IgG secondary antibody (Jackson Immuno Research, West Grove, PA, USA). To ensure that the quantities of protein were similar, the membranes were additionally treated with an antibody against β-actin and Lamin (Santa Cruz Biotechnology, Dallas, TX, USA). Signals were detected using a Super-Signal West Pico Chemiluminescent Substrate (Biogenerica, Pedara, Italy) enhanced chemiluminescence detection system reagent [44]. The relative expression of the protein bands was measured using densitometry and was standardized to β-actin and Lamin levels using Bio-Rad ChemiDoc XRS 2.1.1 software [48]. The blot signal images were input into analysis software (Image Quant TL, v2003, Bio-rad, Segrate, Italy).

### 4.7. Biochemical Analysis

Biochemical analyses were conducted on the hippocampi:

#### 4.7.1. Measurement of SOD Activity

After homogenizing the samples in the Tris buffer, they were centrifuged at 13,000 rpm. The solution was then incubated at 4 °C for 10 min before being centrifuged again. The absorbance of the samples was measured every 60 s for 10 min at 420 nm [57,65].

#### 4.7.2. Measurement of CAT Activity

After homogenizing the samples in the phosphate buffer, hydrogen peroxide was added. Enzyme levels were expressed as CAT activity in U/mg protein, and the absorbance (240 nm) was measured for 0–10 min at 240 min [66].

#### 4.7.3. GSH Levels

A trichloroacetic acid solution was administered after homogenizing the samples with the phosphate buffer. After centrifuging the solution, 5,5′-dithiobis-(2-nitrobenzoic acid) was added. Using a microplate reader, the GSH levels were measured at 412 nm [57].

#### 4.7.4. Measurement of Nitrite Levels

After homogenizing the samples in the phosphate buffer, the Griess reagent was applied. For 30 min, the solution was incubated. At 548 nm, the absorbance was measured [67].

#### 4.7.5. Measurement of MDA

The evaluation of thiobarbituric acid-reactant substances, a suitable indication of lipid peroxidation, was performed on the samples. At 532 nm, the absorbance of the supernatant was measured [68].

#### 4.7.6. Measurement of ROS

After homogenizing the samples in the phosphate buffer, they were exposed to 1 mM dichlorofluorescein diacetate for 10 min at room temperature in the dark (DCFH-DA). The esterase activity used to convert non-fluorescent DCFH-DA to the highly fluorescent product 20,70-dichlorofluorescein (DCF) was used to monitor the presence of peroxides caused by the oxidative burst in the brain [69].

#### 4.7.7. Cytokines and Aβ Measurement

An ELISA kit was used to measure IL-6, TNF-α, IL-1β, and Aβ levels in the hippocampus (Diaclone Research, Biosource Europe, USCN life Sciences; Abcam, Milan, Italy) [35].

### 4.8. Statistical Evaluation

All values are expressed as mean ± standard error of the mean (SD) of N observations. N denotes the number of animals utilized in in vivo studies. One-way ANOVA was used to examine the data, followed by a Bonferroni post hoc test for multiple comparisons. A *p*-value less than 0.05 was regarded as significant.

## 5. Conclusions

In summary, oral treatment with Memophenol^TM^ at a dose of 15 mg/kg, by acting on oxidative stress and inflammatory processes, was able to manage AD features such as behavioral changes related to cognitive functions and memory, phosphorylated Tau levels, and the aberrant overexpression of APP, the accumulation of β-amyloid, and neuronal degeneration. Certainly, taking Memophenol^TM^ cannot cure Alzheimer’s disease, but it is a nutritional supplement that may slow the course of the illness and alleviate symptoms connected with this pathology.

## Data Availability

Based on the rules of our laboratory, the datasets used in the current study are available from the corresponding author (rsiracusa@unime.it) upon reasonable request.
